# Exercise-Based Interventions for the Management of Low Back Pain in Adolescents in School Settings: A Systematic Review

**DOI:** 10.3390/medsci14040473

**Published:** 2026-08-12

**Authors:** Gismael M. Tarão, João Pedro R. Afonso, Alex S. Ribeiro, Marieli R. Stocco, Adriana Bovi, Ludymilla V. Barbosa, André L. Pinto, Claudia S. Oliveira, Karla C. N. de Carvalho, Miriã C. Oliveira, Fernanda S. P. Quirino, Glauber A. Oliveira, Andréia C. R. I. Sampaio, Dunya O. Masri, Luís V. F. Oliveira, Tiago V. Fernandes, Hustênio A. A. Filho, Paolo Capodaglio, Luciana P. Maia, Rodrigo A. C. Andraus

**Affiliations:** 1 Graduate Program in Human Movement and Rehabilitation, Evangelical University of Goiás (UniEVANGÉLICA), Anápolis 75083-515, Brazil; gysmaelmr@hotmail.com (G.M.T.); joaopedro180599@gmail.com (J.P.R.A.); adrianabovil@gmail.com (A.B.); ludymillavgo1234@gmail.com (L.V.B.); andreluciano.motriz@gmail.com (A.L.P.); csantos.neuro@gmail.com (C.S.O.); medkarcri@yahoo.com.br (K.C.N.d.C.); miriacandidaoliveira@gmail.com (M.C.O.); fspfisio@yahoo.com.br (F.S.P.Q.); glauber_br@hotmail.com (G.A.O.); andreia.cristina.izidro@gmail.com (A.C.R.I.S.); omaridunya5@gmail.com (D.O.M.); oliveira.lvf@gmail.com (L.V.F.O.); 2 Faculty of Sports Sciences and Physical Education (FCDEF), University of Coimbra, CIPER, 3004-531 Coimbra, Portugal; alex-silvaribeiro@hotmail.com; 3 Physhiotherapy Department, The State University of Northern Paraná (UENP), Jacarezinho 86400-000, Brazil; marieli.stocco@uenp.edu.br; 4 Department Rehabilitation, Barretos Cancer Hospital, Barretos 14784-400, Brazil; tiago.vieirafernandes@hotmail.com; 5 Health Sciences Graduate Program (PPGCS), Faculty of Medical Sciences (FCMSCSP), Irmandade da Santa Casa de Misericórdia de São Paulo, São Paulo 01221-010, Brazil; hustenio@hotmail.com; 6 Orthopaedic Rehabilitation Unit, Research Laboratory for Biomechanics, Rehabilitation and Ergonomics, Ospedale San Giuseppe, 20123 Milan, Italy; p.capodaglio@auxologico.it; 7 Physical and Rehabilitation Medicine Sector, Department of Biomedical, Surgical and Dental Sciences, University of Milan, 20122 Milan, Italy; 8 Dentistry Graduate Program, Evangelical University of Goiás (UniEVANGÉLICA), Anápolis 75083-515, Brazil; lucianapmaia@gmail.com

**Keywords:** low back pain, adolescents, exercise therapy, school-based intervention, rehabilitation

## Abstract

Background/Objectives: To synthesize the effects of school-based exercise interventions in adolescents with nonspecific low back pain. Methods: PubMed/MEDLINE, Scopus, Web of Science, CENTRAL, PEDro, ScienceDirect, and supplementary sources were searched through 21 May 2026. Randomized and quasi-randomized controlled trials involving adolescents aged 10–19 years and delivering exercise wholly or substantially within schools were eligible. Risk of bias was assessed using Cochrane RoB 1, and findings were synthesized narratively. Results: Of 1935 records, two randomized trials and one quasi-experimental controlled trial (194 allocated participants) were included. Fanucchi et al. reported between-group reductions in past-month pain of 2.2 cm on a 10-cm visual analogue scale (95% CI 1.0–3.5) at 3 months and 2.0 cm (95% CI 0.5–3.5) at 6 months; absolute reductions in 3-month low back pain prevalence were 24% (95% CI 4–41) and 40% (95% CI 18–57), respectively. Jones et al. reported a large effect size for pain severity after 8 weeks (ES = 1.47). Javadipour et al. reported favorable pain and motor-function outcomes, but incomplete and internally inconsistent reporting limited interpretation. No study assessed low-back-pain-related disability using a validated instrument. All studies had high risk of bias in at least one domain, most commonly blinding. Heterogeneity precluded meta-analysis. Conclusions: Limited evidence suggests that school-based exercise may reduce pain intensity and pain prevalence over six months and improve selected physical outcomes in adolescents aged 12–15 years. The small, heterogeneous evidence base and risk of bias preclude conclusions or recommendations regarding modality or dosage.

## 1. Introduction

Low back pain (LBP) is one of the leading causes of disability worldwide and represents a substantial public health burden [1,2]. Although its global burden has been characterized predominantly in adults, LBP is also common in children and adolescents, with prevalence increasing throughout adolescence [3,4]. Reported prevalence estimates range from 7% to 72%, reflecting differences in age groups, case definitions, and recall periods across studies [5,6]. LBP during adolescence may recur, restrict participation in physical activities, impair quality of life, and be associated with symptoms persisting into adulthood [3,7].

When symptoms cannot be attributed to a recognizable specific pathology, the condition is classified as nonspecific low back pain (NSLBP) [3,4]. Exercise is commonly used as a non-pharmacological intervention for NSLBP and may contribute to improvements in strength, mobility, motor control, physical function, and pain-related outcomes. However, evidence specific to adolescents remains limited. Previous systematic reviews of conservative interventions have generally combined heterogeneous populations, multiple therapeutic approaches, and different intervention settings, making it difficult to determine the effects of exercise specifically for adolescents with established NSLBP [3,4,6,8]. Furthermore, findings derived from adults cannot be directly extrapolated to adolescents because of developmental, clinical, and contextual differences between these populations.

Schools may provide a practical setting for delivering exercise interventions because they allow repeated access to adolescents within a structured environment and may facilitate supervised participation. Nevertheless, school-based studies may address substantially different questions, including primary prevention in asymptomatic populations and treatment in adolescents with existing symptoms. These populations should be distinguished because evidence from prevention programs does not directly establish treatment effects in adolescents with NSLBP. School-based exercise programs also vary considerably in modality, frequency, intensity, duration, supervision, and outcome assessment, and their therapeutic effects have not been clearly established.

Therefore, this systematic review aimed to synthesize the effects of exercise interventions delivered wholly or substantially within school settings for adolescents aged 9–16 years with NSLBP. Particular attention was given to pain intensity, pain prevalence, and LBP-related disability, while physical and motor outcomes, intervention characteristics, adherence, and adverse events were also examined. The review further sought to determine whether the available evidence supports conclusions regarding specific exercise modalities or dosages.

## 2. Materials and Methods

### 2.1. Study Design

This systematic review was conducted in accordance with the Cochrane Handbook for Systematic Reviews of Interventions [9,10] and reported according to the Preferred Reporting Items for Systematic Reviews and Meta-Analyses (PRISMA 2020) statement [11]. The review protocol was developed in accordance with the PRISMA-P guidelines [12]. The review was retrospectively registered in the International Prospective Register of Systematic Reviews (PROSPERO; CRD420261297319).

The review question was structured according to the PICOT framework. The population comprised adolescents aged 10–19 years with existing nonspecific low back pain (NSLBP) who were enrolled in formal primary, middle, or secondary education. Eligible interventions were structured exercise programs in which exercise was the principal therapeutic component and which were delivered wholly or substantially within the school setting. Comparators included no intervention, usual care, education alone, or an alternative exercise intervention. Outcomes of primary interest were pain intensity, pain prevalence or recurrence, and LBP-related disability. Secondary outcomes included quality of life, physical function, muscle strength, flexibility, posture, kinesiophobia, school absenteeism, adherence, and adverse events. A minimum intervention duration of four weeks was required to exclude single-session or very short-term experimental interventions and to ensure repeated exposure to a structured therapeutic program.

Several amendments were made during the conduct of the review and are reported here for transparency. The published protocol proposed the inclusion of non-randomized studies and the use of ROBINS-I. The final eligibility criteria were revised to include randomized controlled trials, quasi-randomized controlled trials, and controlled quasi-experimental studies, thereby prioritizing controlled evidence directly addressing intervention effects. Risk of bias was assessed using the original Cochrane Risk of Bias tool (RoB 1), because two included studies were randomized trials and the third reported random allocation to intervention and control groups. Web of Science, PEDro, ScienceDirect, and supplementary sources were added to improve search coverage. The outcome framework was also expanded beyond pain intensity to include pain prevalence or recurrence, LBP-related disability, and relevant physical outcomes reported by the eligible studies. These amendments did not alter the central population, therapeutic exercise focus, or school-setting requirement of the review.

### 2.2. Eligibility Criteria

Randomized controlled trials, quasi-randomized controlled trials, and prospective controlled quasi-experimental studies were eligible. Studies were required to include adolescents aged 10–19 years with existing NSLBP who were enrolled in a formal primary, middle, or secondary school. For this review, formal school education referred to mainstream educational settings providing primary or secondary education, irrespective of whether the institution was public or private. This definition was used to distinguish school-based delivery from interventions conducted exclusively in clinical, rehabilitation, residential, university, or laboratory settings.

NSLBP was operationally defined as pain located between the lower rib margin and the gluteal region that could not be attributed to a specific underlying disease, structural spinal pathology, or other identifiable cause [1,3]. Because diagnostic procedures were not standardized across the eligible trials, studies were accepted when existing LBP was identified using predefined criteria established by the original investigators, including clinical assessment, pain questionnaires, or prespecified self-report screening. The diagnostic or screening procedure used in each study was extracted and reported in the study characteristics table.

Exercise was required to constitute the principal active component of the intervention. Eligible programs included strengthening, trunk or lumbopelvic stabilization, motor-control exercises, stretching, flexibility exercises, Pilates, postural exercises, functional training, and structured physical activity. General physical activity or physical education programs were eligible only when they comprised a predefined and structured exercise program intended to treat adolescents with existing LBP. Health or postural education could be included as a co-intervention, provided that exercise remained the main therapeutic component.

A school-based intervention was defined as a structured exercise program in which all supervised sessions, or a substantial component of them, were delivered at school during or in connection with the school routine. Additional home-exercise components were permitted when the school remained a central site of intervention delivery. Studies in which schools were used solely for recruitment, screening, or outcome assessment, while the intervention was delivered entirely in a clinic, university laboratory, research center, or other external setting, were excluded.

Eligible comparators included no intervention, usual care, waiting-list control, education alone, or another exercise intervention. Studies were required to report at least one LBP-related outcome, including pain intensity, pain prevalence or recurrence, or LBP-related disability. Secondary outcomes were extracted when available, but studies were not required to report every outcome of interest.

For studies involving mixed populations or multiple participant subgroups, only data from adolescents meeting the predefined criteria for NSLBP were eligible. Mixed-population studies were included only when data for the relevant NSLBP subgroup could be extracted separately. Participants without LBP who received interventions exclusively intended for primary prevention were excluded from the evidence synthesis.

Studies were excluded if participants had specific spinal conditions or red flags, including spinal trauma or fracture, neoplasms, infections, inflammatory diseases, previous spinal surgery, neurological disorders, or severe structural deformities. Uncontrolled intervention studies, observational studies, case reports, case series, reviews, study protocols, editorials, letters, dissertations, theses, conference abstracts, and reports with insufficient information to determine eligibility or extract relevant outcomes were also excluded. The intervention was required to last at least four weeks.

### 2.3. Information Sources

A comprehensive search was conducted in MEDLINE via PubMed, Scopus, Web of Science, the Cochrane Central Register of Controlled Trials (CENTRAL), the Physiotherapy Evidence Database (PEDro), and ScienceDirect. These sources were selected to provide complementary coverage of biomedical literature, multidisciplinary citation indexes, controlled trials, physiotherapy-specific evidence, and additional health and life-sciences publications, in accordance with the recommendations of the Cochrane Handbook [10].

Supplementary searches were conducted in Google Scholar, the OpenGrey archive, and ProQuest Dissertations & Theses Global. ClinicalTrials.gov and the World Health Organization International Clinical Trials Registry Platform (WHO ICTRP) were also searched for ongoing, completed, or unpublished trials. Backward citation searching was performed by screening the reference lists of the included studies and relevant systematic reviews, and forward citation searching was used to identify additional potentially eligible reports.

Grey-literature sources, dissertations, theses, and trial registries were examined to identify unpublished studies and trace any corresponding eligible full-text publications. However, dissertations, theses, registry records, and conference abstracts were not themselves eligible as final reports under the predefined publication criteria. No restrictions were imposed according to language, country, or publication date. The final electronic searches were conducted on 21 May 2026. Complete database-specific strategies, including search terms, Boolean operators, field tags, adaptations, and limits, are presented in Supplementary Material Table S1.

### 2.4. Search Strategy

The search strategy combined controlled vocabulary terms, when available, and free-text terms related to four core concepts: nonspecific low back pain, adolescents, exercise-based interventions, and school settings. Synonyms within each concept were combined using the Boolean operator “OR”, and the four concepts were combined using “AND”. No language, country, publication-date, or study-status restrictions were applied.

The complete PubMed/MEDLINE search strategy was:

((“Low Back Pain”[Mesh] OR “low back pain” OR lumbago OR lumbalgia OR “lumbar pain” OR “back pain” OR “nonspecific low back pain” OR “non-specific low back pain” OR NSLBP) AND (“Adolescent”[Mesh] OR “Child”[Mesh] OR adolescent* OR adolescenc* OR teenager* OR teen* OR youth OR student* OR schoolchild* OR “school-aged”) AND (“Exercise Therapy”[Mesh] OR “Exercise”[Mesh] OR “Motor Activity”[Mesh] OR exercis* OR “therapeutic exercise” OR “physical activity” OR strengthening OR “resistance training” OR “stabilization exercise” OR “stabilization training” OR “core training” OR Pilates OR stretching OR “flexibility exercise” OR “motor control exercise” OR “postural exercise” OR rehabilitation OR physiotherap* OR kinesiotherap* OR “training program*”) AND (“Schools”[Mesh] OR “School Health”[Mesh] OR school* OR “school-based” OR “school setting” OR classroom OR “physical education” OR PE)).

The strategy was adapted to the syntax, indexing system, and search interface of Scopus, Web of Science, CENTRAL, PEDro, and ScienceDirect. Supplementary searches used predefined combinations of terms related to low back pain, adolescents, exercise, and schools. Google Scholar results were screened manually in relevance-ranked order. ClinicalTrials.gov was searched using “Low Back Pain” as the condition and “adolescent”, “exercise”, and “school” as additional terms, whereas WHO ICTRP was searched using combinations of “low back pain”, “adolescent”, and “exercise”. Reference lists of included studies and relevant systematic reviews were screened, and forward and backward citation searching was performed for the eligible reports.

The final electronic and supplementary searches were conducted on 21 May 2026. Complete source-specific strategies, including search terms, field tags, Boolean operators, adaptations, filters or limits, and search dates, are provided in Supplementary Material Table S1.

### 2.5. Eligible Studies

All records retrieved from the electronic and supplementary searches were imported into EndNote (Clarivate) for reference management. Potential duplicate records were identified using EndNote’s automated duplicate-detection function. All records identified as possible duplicates were subsequently reviewed and confirmed manually before removal. The deduplicated records were then uploaded to Rayyan [13]. No automation tool or machine-learning classifier was used to determine study eligibility; all title-and-abstract and full-text exclusions were based on independent reviewers’ judgments.

Two reviewers (G.M.T. and J.P.R.A.) independently screened titles and abstracts. Screening decisions were masked so that each reviewer remained unaware of the other reviewer’s judgments until the screening stage was completed. Reports considered potentially eligible by either reviewer were retrieved in full text and independently assessed against the predefined eligibility criteria by the same reviewers.

Disagreements at either stage were initially resolved through discussion and consensus. When consensus could not be reached, a third reviewer (M.R.S.) made the final decision. Multiple reports relating to the same study were linked and treated as a single study whenever appropriate. Reasons for exclusion were recorded for all reports excluded after full-text assessment and are presented by category in the PRISMA 2020 flow diagram and individually in the Supplementary Tables S1–S3 of full-text exclusions.

Rayyan was used to organize and support the screening process; however, no automation tool, machine-learning classifier, or automated eligibility rule was used to exclude records or replace reviewers’ judgments.

### 2.6. Data Extraction

Data were independently extracted by two reviewers (G.M.T. and J.P.R.A.) using separate standardized extraction forms developed in accordance with the Cochrane Handbook for Systematic Reviews of Interventions [14]. The completed forms were compared, and discrepancies were resolved through discussion and consensus or, when necessary, consultation with a third reviewer (M.R.S.).

The extracted information included study identification, publication year, country, study design, recruitment setting, eligibility and diagnostic criteria, participant characteristics, sample sizes at screening, allocation, follow-up, and analysis, age, sex, and baseline pain characteristics. Intervention data included exercise modality, therapeutic components, school setting, supervision, co-interventions, program duration, session frequency, session duration, intensity or progression when reported, home-exercise components, adherence, and compliance. Comparator characteristics were extracted in equivalent detail.

For each outcome, the reviewers extracted the measurement instrument, outcome definition, recall period, assessment time point, number of participants analyzed, group-level summary data, between-group estimates, confidence intervals, effect sizes, and statistical test results, as available. Attrition, reasons for withdrawal, missing outcome data, adverse events, funding sources, and reported conflicts of interest were also recorded.

Effect estimates and 95% confidence intervals were extracted directly from the original reports. When a study reported only group means, standard deviations, proportions, or test statistics, these data were presented descriptively. Missing standard deviations, confidence intervals, or between-group effect estimates were not imputed or reconstructed when the information required for reliable calculation was unavailable. Internally inconsistent results were checked against the full-text report and were not recalculated when the correct value could not be determined. Corresponding authors were contacted when clarification or additional information was required; unresolved information was reported as unavailable or unclear.

### 2.7. Quality Assessment

Two reviewers (G.M.T. and J.P.R.A.) independently assessed risk of bias using the original Cochrane Risk of Bias tool for randomized trials (RoB 1) [15]. The following domains were evaluated: random sequence generation, allocation concealment, blinding of participants and personnel, blinding of outcome assessment, incomplete outcome data, selective outcome reporting, and other potential sources of bias. Each domain was classified as “low risk”, “high risk”, or “unclear risk” of bias, with a supporting justification based on the information reported in each article.

Although Javadipour et al. described their study as quasi-experimental, the authors reported that participants were randomly allocated to intervention and control groups. RoB 1 was therefore applied consistently to all three included studies. The absence of sufficient information regarding the method of sequence generation, allocation concealment, or other methodological procedures was reflected in the corresponding domain judgments rather than interpreted as evidence of adequate methods.

Disagreements were resolved through consensus or consultation with a third reviewer (M.R.S.). Risk-of-bias judgments were entered into Review Manager 5.4.1, which was used to generate the risk-of-bias summary and risk-of-bias graph.

### 2.8. Data Synthesis

A structured narrative synthesis was conducted in accordance with the Synthesis Without Meta-analysis reporting guideline (SWiM) [16]. Studies were grouped according to predefined outcome domains: pain intensity; low back pain prevalence or recurrence; low-back-pain-related disability; physical and motor outcomes; adherence; and adverse events. Physical outcomes representing different constructs—including flexibility, spinal or hip mobility, muscular endurance, balance, posture, and lumbopelvic stability—were analyzed separately and were not combined into a single functional outcome.

Within each outcome domain, the synthesis prioritized the direction and magnitude of between-group effects, 95% confidence intervals when available, reported effect sizes, consistency of the findings, risk of bias, and clinical relevance. Findings based on directly reported between-group comparisons were given greater interpretive weight than findings based solely on within-group changes or incompletely reported statistical analyses. Statistical significance was treated as complementary information and was not used for vote counting or as the sole criterion for determining the presence or importance of an intervention effect.

When studies reported more than one assessment time point, post-intervention and follow-up results were presented separately. Results obtained at different follow-up periods were not pooled. Effect estimates were retained in the metrics reported by the original studies, including between-group mean differences, standardized effect sizes, absolute risk differences, proportions, and corresponding 95% confidence intervals.

To contextualize the magnitude of changes in pain intensity, an improvement of approximately 1 point on an 11-point numerical rating scale, or 12.5% relative to baseline, was considered a pediatric and adolescent chronic-pain benchmark [17]. A difference of 10 mm on a 100-mm visual analogue scale was also considered as a supplementary pediatric benchmark [18]. Because these thresholds were derived from within-person changes in broader pediatric pain populations rather than specifically from adolescents with NSLBP, they were not applied as rigid cutoffs for interpreting between-group superiority. No threshold for low-back-pain-related disability was applied because none of the included studies assessed disability using a validated disability-specific instrument. The clinical relevance of other physical and motor outcomes was interpreted cautiously because validated adolescent-specific minimum important differences were generally unavailable.

Meta-analysis was considered separately for each main outcome but was not performed. Pain-intensity data differed in the pain construct assessed, recall period, assessment time point, participant subgroup, and form of effect reporting. Although standardized mean differences could address differences between numerical scales, they would not resolve clinically important differences in outcome definitions or follow-up periods. Low back pain prevalence was reported by only one eligible study, disability was not measured with a validated instrument, and physical or motor outcomes represented distinct constructs assessed using different procedures. In addition, reporting inconsistencies in Javadipour et al. prevented reliable calculation of comparable effect estimates. The studies were therefore considered insufficiently comparable for statistically meaningful pooling.

A formal certainty-of-evidence assessment using GRADE was not undertaken, and no Summary of Findings table was prepared; consequently, no formal certainty categories were assigned to the outcome-level evidence.

### 2.9. Reporting Bias Assessment

Formal assessment of publication bias or small-study effects using funnel plots or statistical tests for funnel-plot asymmetry, such as Egger’s regression test, was not performed because only three studies were included and no meta-analysis was undertaken [19]. Such methods would be underpowered and potentially misleading with this number of studies. Potential reporting and dissemination biases were instead considered qualitatively through searches of grey-literature sources and trial registries and through the selective-reporting domain of the RoB 1 assessment. Nevertheless, the possibility of publication bias could not be excluded.

## 3. Results

### 3.1. Study Selection

The database and registry searches identified 1935 records. EndNote’s automated duplicate-detection function flagged 189 potentially duplicate records. All records identified by the software were manually reviewed, and additional manual verification was performed. In total, 196 duplicate records were removed, leaving 1739 records for title and abstract screening.

Of these, 1693 records were excluded because they did not meet the predefined eligibility criteria. The remaining 46 reports were sought for retrieval, and all were obtained and assessed in full text. After full-text assessment, 43 reports were excluded because of an ineligible population (*n* = 8), absence of an eligible low back pain diagnosis or outcome (*n* = 7), intervention not based on exercise (*n* = 5), intervention not delivered in a school setting (*n* = 9), ineligible study design (*n* = 6), exclusive focus on primary prevention (*n* = 5), or absence of an appropriate comparator group (*n* = 3).

Supplementary searches identified eight additional records through websites (*n* = 2) and citation searching (*n* = 6). All corresponding reports were retrieved and assessed in full text. These reports were excluded because the intervention was not conducted in a school setting (*n* = 4), the population was ineligible (*n* = 1), the intervention exclusively addressed primary prevention (*n* = 2), or no eligible low back pain diagnosis or outcome was reported (*n* = 1).

**Figure 1:**
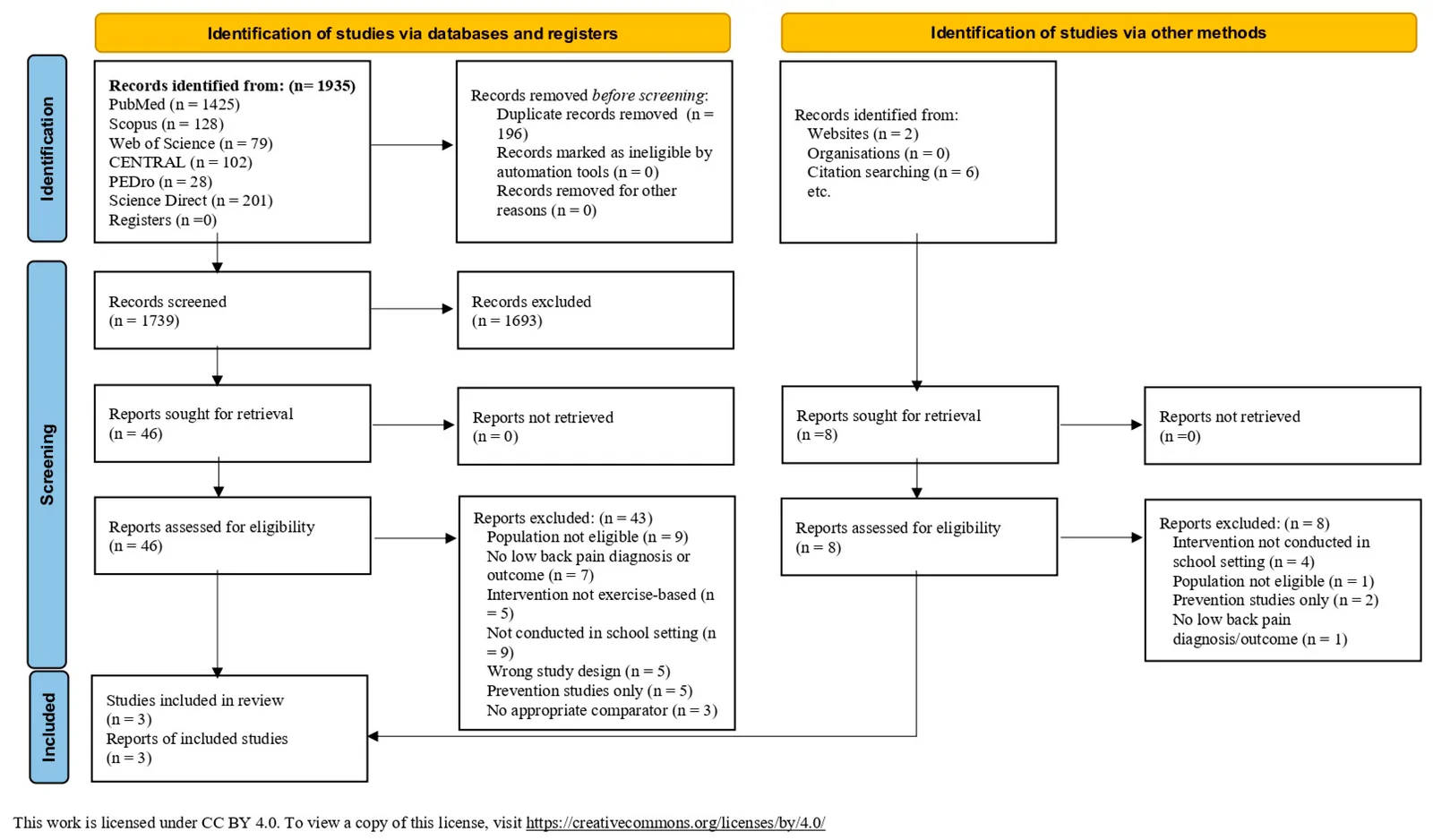
PRISMA 2020 flow diagram of study selection. Source: [12].

Ultimately, three reports, each representing a distinct study, met the eligibility criteria and were included in the narrative synthesis: two randomized controlled trials and one controlled quasi-experimental study [20,21,22]. The study-selection process is presented in the PRISMA 2020 flow diagram (Figure 1). The studies excluded after full-text assessment and the specific reason for each exclusion are presented in Supplementary Table S1.

### 3.2. Characteristics of the Included Studies

The three included studies were published between 2007 and 2021 and were conducted in the United Kingdom, South Africa, and Iran [20,21,22]. Two studies were randomized controlled trials [20,21], whereas Javadipour et al. described their study as quasi-experimental with pre- and post-intervention assessments and control groups, although the authors reported that participants were randomly divided among the four study groups [22].

Across the three studies, 194 participants were allocated to intervention or control conditions. The available final analyses comprised 184 participants: 54 in Jones et al. [14], 70 at the six-month assessment in Fanucchi et al. [15], and 60 in the analysis tables of Javadipour et al. [22]. Participants were aged 12–15 years, which represents a narrower age range than the 10–19-year eligibility range established for this review.

Jones et al. screened 405 students from Grades 9 and 10 in two secondary schools and identified 88 adolescents with recurrent NSLBP [20]. Sixty-two participants consented and were allocated in matched pairs to exercise rehabilitation (*n* = 31) or control (*n* = 31). Four participants withdrew, two from each group, and their four matched partners were also excluded from the analysis. Consequently, 54 participants were analyzed: 27 in each group. The mean age of the analyzed participants was 14.6 years.

Fanucchi et al. screened 99 students from Grades 6 and 7 in two government primary schools and randomized 72 students aged 12–13 years who had reported LBP during the preceding three months [21]. Thirty-nine were allocated to the exercise group and 33 to the control group. At three months, 39 intervention and 32 control participants were assessed; one control participant was lost because she left the school. At six months, 38 intervention and 32 control participants were assessed; one intervention participant was excluded after sustaining a serious back injury outside the study. Thus, 70 of the 72 randomized participants were available at the final follow-up.

Javadipour et al. purposefully selected 60 girls aged 12–15 years with chronic NSLBP from schools in Lahijan, Iran [22]. Participants were reported to have symptoms lasting longer than three months, specialist confirmation, and a score greater than 50 on a measure described by the authors as the Quebec Back Pain Test. Participants were divided into four groups of 15 according to intervention status and the presence or absence of lumbar hyperlordosis. No withdrawals or losses to follow-up were reported, and the analysis tables maintained *n* = 15 and df = 14 for each group, suggesting that all 60 participants were analyzed. Nevertheless, the participant description stated that 15 students had hyperlordosis and 30 did not, accounting for only 45 participants. This internal inconsistency prevented confirmation of the exact distribution between the lordosis strata.

The methods used to identify NSLBP differed among the studies. Jones et al. used a standardized questionnaire followed by an interview to identify recurrent NSLBP characterized by repeated acute episodes [20]. Fanucchi et al. included students who reported LBP during the preceding three months and excluded specific spinal, neurological, and systemic conditions [21]. Javadipour et al. included girls with specialist-confirmed chronic NSLBP and applied additional pain and postural criteria [22].

All three studies reported pain-related outcomes. Additional outcomes included pain prevalence, interference with physical activity, school absenteeism, flexibility, spinal and hip mobility, neural mobility, lumbopelvic stability, proprioception, trunk-muscle endurance, lumbar lordosis, static and dynamic balance, and psychological well-being. Jones et al. assessed activity restriction and school absenteeism as indirect indicators of pain-related disability [20]. Javadipour et al. used a 25-item Quebec measure scored from 0 to 100, which the authors described as a measure of pain intensity [22]. However, the format and scoring correspond to the Quebec Back Pain Disability Scale; therefore, this outcome was treated separately because its construct was inconsistently identified in the original report. None of the studies used an unambiguously identified and appropriately interpreted adolescent-specific measure of LBP-related disability. The main characteristics and participant flow of the included studies are summarized in Table 1.

### 3.3. Characteristics of the Interventions

All included interventions lasted eight weeks and were delivered within school settings, but their frequency, session duration, exercise components, progression, supervision, and additional home components differed substantially. The interventions are summarized according to the Frequency, Intensity, Time, and Type principles in Table 2.

Jones et al. implemented a multimodal exercise-rehabilitation program consisting of strength, flexibility, and aerobic exercises [20]. Participants completed two approximately 30-min structured group sessions per week, totaling 16 planned sessions. The program was time-contingent and progressive and incorporated pain-relieving, reconditioning, and progressive exercises. All participants followed the same standardized progression, prescribed exercises, and repetitions. Home exercises at a pain-relieving level were encouraged, although the supervising professional and the specific external loads or intensity targets were not reported.

Fanucchi et al. provided eight supervised exercise classes during school hours, corresponding to one class per week [21]. Each class lasted approximately 40–45 min and was supervised by a registered physiotherapist. The program emphasized spinal and pelvic stabilization, core-muscle control, flexibility, posture, and spinal alignment. It began with a 10–15-min educational session, and the educational principles were reinforced throughout the program. Exercises were progressively advanced over the eight weeks, although quantitative intensity or loading parameters were not reported. Participants also received a weekly home-exercise program comprising exercises previously taught during the supervised classes.

Javadipour et al. delivered three 45-min sessions per week in the school hall, totaling 24 planned sessions [22]. The program comprised a warm-up, core-stability and stretching exercises, and a cool-down. Exercises targeted the abdominal, lumbar, trunk-flexor, trunk-extensor, lateral trunk, piriformis, and lumbar-pelvic muscle groups. Progression was achieved by reducing rest intervals and increasing the number of sets, repetitions, and contraction duration. Strengthening exercises were performed in three sets, with contraction duration progressing from 10 s to 35 s. Stretching exercises were also performed in three sets, with repetitions increasing from 10 to 30. Sessions were supervised by the researcher.

The control groups in Fanucchi et al. and Javadipour et al. received no exercise intervention, whereas the control group in Jones et al. continued its usual daily activities and was offered the exercise program after completion of the study period [20,21,22]. Because all interventions were multimodal and no study directly compared different exercise modalities or dosages, the available studies do not permit identification of a superior exercise component or protocol.

### 3.4. Risk-of-Bias Assessment

The risk of bias of the included studies was assessed across the seven domains of the original Cochrane Risk of Bias tool (RoB 1) [15]. No study was judged to be at low risk of bias across all domains. Blinding of participants and personnel was judged to be at high risk in all three studies because participants were necessarily aware of whether they received the exercise intervention.

Fanucchi et al. had the most favorable methodological profile [21]. Random-sequence generation and allocation concealment were adequately described, outcome assessment was blinded, attrition was low and explained, and no important concerns were identified regarding selective reporting or other sources of bias. The study was therefore judged to be at low risk in six domains and at high risk only for blinding of participants and personnel.

Jones et al. did not report the method used to generate the random sequence or sufficient procedures to confirm allocation concealment [20]. The post-intervention assessor was aware of group allocation, resulting in a high-risk judgment for blinding of outcome assessment. Four participants withdrew, and their four matched partners were subsequently excluded from the analysis; reasons for withdrawal were not reported, post-intervention data were unavailable, and an intention-to-treat analysis was not performed. Incomplete outcome data were therefore judged to be at high risk. Selective reporting was judged to be unclear because no prospective protocol or trial registration was identified.

Javadipour et al. reported that participants were randomly divided among four groups but did not describe the method of sequence generation or allocation concealment [22]. Blinding of outcome assessment and procedures for handling missing data were not adequately reported. Selective reporting was judged to be unclear because no prospective protocol or trial registration was identified. Other bias was judged to be high because the participant description accounted for only 45 of the 60 participants presented in the analysis tables, and several values in the outcome tables were internally inconsistent with the reported statistical results.

**Figure 2:**
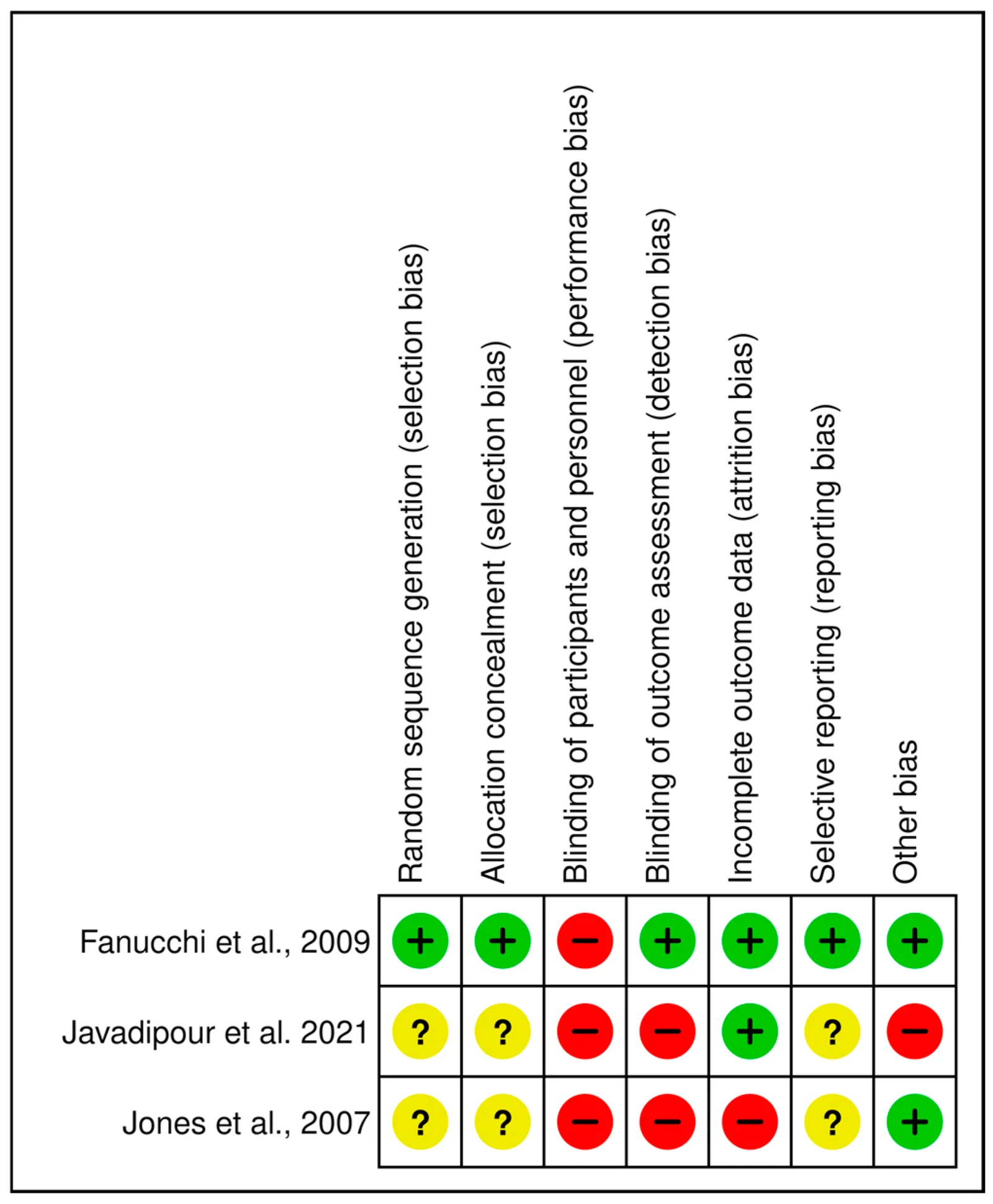
Risk-of-bias summary: review authors’ judgments for each risk-of-bias domain across the included studies. The symbols indicate the following judgments: +, low risk of bias; −, high risk of bias; and ?, unclear risk of bias [20,21,22].

**Figure 3:**
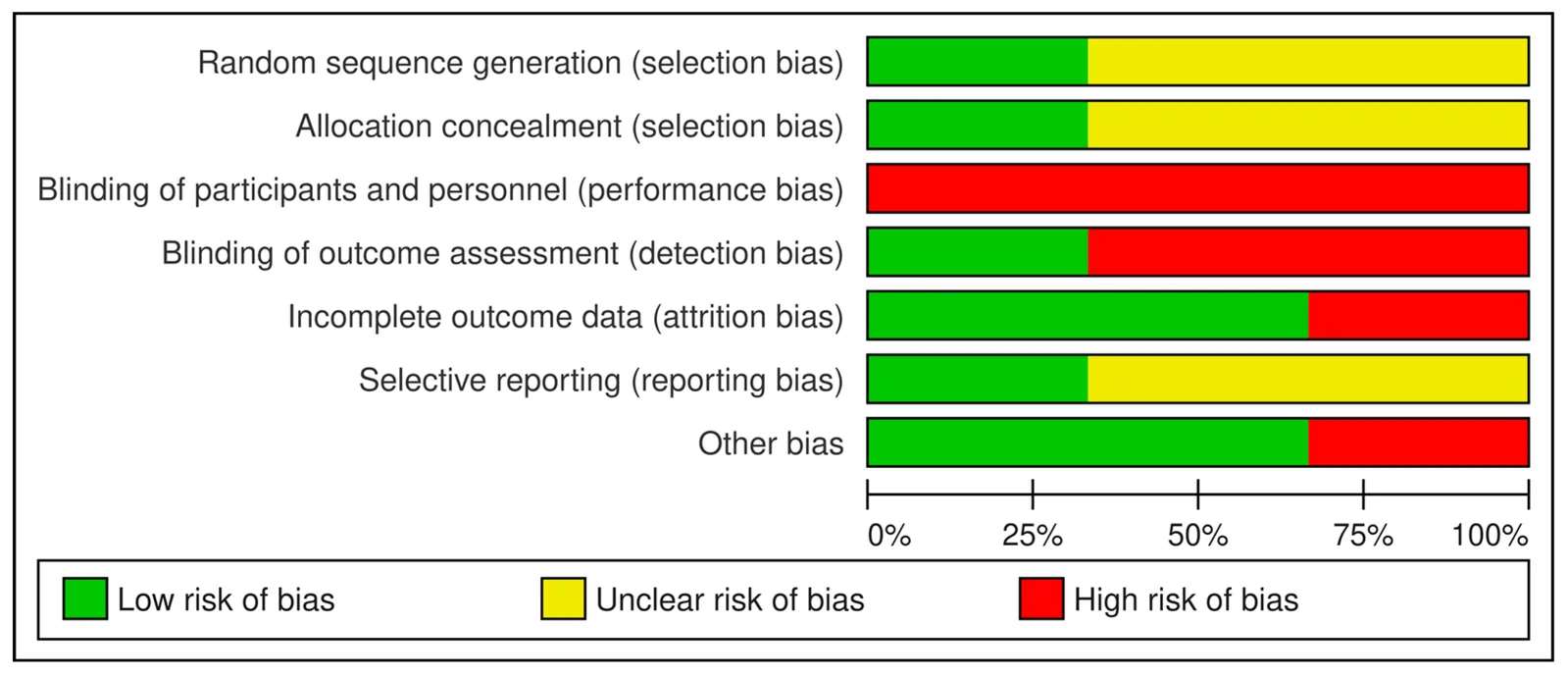
Risk-of-bias graph: percentage distribution of judgments across risk-of-bias domains for all included studies.

The complete domain-level judgments are presented in the risk-of-bias summary and graph in Figure 2 and Figure 3.

### 3.5. Effects of Exercise Interventions on Low Back Pain

The effects were synthesized separately for pain intensity, pain prevalence or frequency, LBP-related disability and participation, and physical and motor outcomes. The main quantitative findings are presented in Table 3. Direct between-group estimates with 95% confidence intervals were prioritized when reported. When these estimates were unavailable, group-specific changes and the statistical interaction reported by the original study are presented.

#### 3.5.1. Pain Intensity

Jones et al. and Fanucchi et al. assessed pain intensity using an unambiguously identified numerical pain scale or visual analogue scale [20,21].

In Jones et al., mean pain intensity in the exercise group decreased from 6.5 ± 1.2 to 3.7 ± 1.3 points after eight weeks, whereas the control-group mean increased from 5.3 ± 2.3 to 6.0 ± 1.5 points [20]. The reported change was −2.73 points (95% CI −3.16 to −2.29) in the exercise group and 0.70 points (95% CI 0.04 to 1.36) in the control group. The group-by-time interaction was reported as *p* = 0.001, with an effect size of 1.47. A direct between-group mean difference and corresponding confidence interval were not reported.

Fanucchi et al. distinguished pain experienced during the preceding month from current pain [21]. For pain during the preceding month, the between-group difference in change favored the exercise group by −2.2 cm on the 10-cm VAS at Month 3 (95% CI −3.5 to −1.0) and by −2.0 cm at Month 6 (95% CI −3.5 to −0.5). These differences exceeded the 1-cm pediatric VAS benchmark used for contextual interpretation, although this threshold was not developed specifically for between-group effects in adolescents with NSLBP.

In contrast, between-group differences for current pain were −1.2 cm at Month 3 (95% CI −2.6 to 0.2) and 0.3 cm at Month 6 (95% CI −1.3 to 1.9). Both confidence intervals crossed the null value. Thus, the effect observed by Fanucchi et al. depended on the pain construct and recall period: differences were observed for pain during the preceding month but not for current pain.

The Quebec measure used by Javadipour et al. was not combined with these pain-intensity results because its 25-item format corresponded to a disability-related construct, despite being described as pain intensity by the study authors [22].

#### 3.5.2. Pain Prevalence and Frequency

Fanucchi et al. was the only study to report three-month LBP prevalence [21]. At Month 3, 26 of 39 participants in the exercise group (67%) and 29 of 32 participants in the control group (91%) reported LBP during the preceding three months. The absolute risk reduction was 24% (95% CI 4% to 41%). At Month 6, the corresponding proportions were 16 of 38 participants (42%) and 26 of 32 participants (81%), resulting in an absolute risk reduction of 40% (95% CI 18% to 57%).

Jones et al. assessed the number of days on which LBP was experienced during a seven-day diary period [20]. The exercise-group mean decreased from 3.4 ± 1.5 to 2.9 ± 1.3 days, whereas the control-group mean increased from 3.5 ± 1.8 to 3.7 ± 1.2 days. The group-by-time interaction was *p* = 0.036, with an effect size of 0.39. Because Jones et al. prespecified *p* < 0.01 as the statistical threshold, this finding did not meet the study’s criterion for statistical significance.

None of the included studies directly assessed recurrence using a predefined recurrence definition or a sufficiently long symptom-free observation period. Consequently, no conclusion regarding LBP recurrence was drawn.

#### 3.5.3. LBP-Related Disability and Participation

Jones et al. assessed the number of occasions on which LBP prevented participation in physical activity and the number of school absences during a seven-day diary period [20]. Occasions of prevented physical activity decreased from 1.1 ± 1.1 to 0.2 ± 0.5 in the exercise group and changed from 0.7 ± 1.0 to 0.8 ± 0.9 in the control group. The reported changes were −0.96 occasions (95% CI −1.35 to −0.58) and 0.07 occasions (95% CI −0.24 to 0.39), respectively. The group-by-time interaction was *p* = 0.001, with an effect size of 0.99.

School absenteeism was low at baseline and did not change appreciably. The exercise-group change was −0.04 absences (95% CI −0.11 to 0.04), compared with 0.00 absences in the control group (95% CI −0.15 to 0.15). The group-by-time interaction was *p* = 0.661, with an effect size of 0.27.

Javadipour et al. used a 25-item Quebec measure scored from 0 to 100, with lower scores indicating fewer pain-related limitations [22]. Although the study described this outcome as pain intensity, its structure corresponds to a disability-related measure. Among participants with hyperlordosis, the reported mean decreased from 67.26 to 9.53 in the exercise group, whereas it changed from 56.26 to 59.06 in the control group. Among participants without hyperlordosis, the corresponding means changed from 59.80 to 14.60 and from 59.20 to 58.53. The between-group analyses were reported as *p* = 0.001 for both postural strata. However, the authors did not report between-group effect estimates or confidence intervals, and the inconsistent identification of the measured construct limits direct comparison with the other pain-related outcomes.

Therefore, none of the included studies provided an unambiguous assessment of LBP-related disability using an appropriately identified and interpreted adolescent-specific disability instrument.

#### 3.5.4. Physical and Motor Outcomes

Jones et al. reported group-by-time interactions for all five physical outcomes examined [20]. The exercise group demonstrated improvements in sit-and-reach performance, hip range of motion, trunk-muscle endurance, lateral spinal flexion, and lumbar sagittal mobility, whereas the control group showed little change or deterioration. Reported effect sizes were 0.91 for sit-and-reach performance, 0.91 for hip range of motion, 0.70 for trunk-muscle endurance, 0.52 for lateral spinal flexion, and 0.82 for lumbar sagittal mobility. The group-by-time interaction was *p* = 0.001 for each outcome. Changes in these physical outcomes were not significantly correlated with changes in pain intensity, with correlation coefficients ranging from r = 0.08 to r = 0.23.

Fanucchi et al. reported between-group improvements at Month 3 in hamstring and iliopsoas flexibility and passive straight-leg-raise performance [21]. The between-group differences in hamstring flexibility were 8° for the right leg (95% CI 4° to 13°) and 15° for the left leg (95% CI 11° to 19°). Corresponding differences in iliopsoas flexibility were 6° (95% CI 2° to 11°) and 10° (95% CI 5° to 15°). Passive straight-leg-raise differences were 16° for the right leg (95% CI 10° to 20°) and 14° for the left leg (95% CI 6° to 22°).

At Month 6, the between-group differences for passive straight-leg raise remained 13° for the right leg (95% CI 6° to 20°) and 10° for the left leg (95% CI 4° to 16°). Differences in muscle flexibility were less consistent: the left hamstring difference remained 6° (95% CI 2° to 10°), whereas the confidence intervals for the right hamstring and right iliopsoas crossed the null value. No between-group differences were identified for rectus femoris flexibility, active straight-leg-raise performance as a measure of lumbar stability, or lumbosacral proprioception. Fanucchi et al. also found no between-group differences in psychological well-being at Month 3 or Month 6.

Javadipour et al. reported between-group differences in lumbar lordosis angle and trunk extensor, flexor, and lateral-flexor endurance in participants with and without hyperlordosis [22]. For static and dynamic balance, between-group differences were reported only in participants without hyperlordosis (*p* = 0.001 for both outcomes). Static and dynamic balance did not differ between the exercise and control groups among participants with hyperlordosis (*p* = 0.477 and *p* = 0.541, respectively). Confidence intervals and standardized effect sizes were not reported. Because several descriptive values in the outcome tables were internally inconsistent with the reported test statistics, additional effect estimates were not reconstructed.

#### 3.5.5. Adherence, Acceptability, and Adverse Events

Jones et al. reported an overall compliance rate of 88% [20]. Every participant retained in the exercise group attended at least 12 of the 16 planned sessions. Home pain-relieving exercises were encouraged, but adherence to the home component was not quantified. No participant was prevented from continuing an exercise session because of NSLBP; however, the causes of individual session absences were not determined, and the study did not describe a systematic adverse-event assessment.

In Fanucchi et al., most participants attended all eight supervised classes, while five participants (13%) missed more than one class [21]. Approximately one-third reported completing the home program at least three times per week, 20% completed it fewer than two times per week, and one participant did not perform any home exercises. Regarding acceptability, 85% reported enjoying the classes, 97% felt that the exercises helped them feel better, and 81% felt that the program made their backs stronger. One participant sustained a serious back injury outside the study and was excluded before the six-month assessment. The event was explicitly reported as occurring outside the intervention, and no other adverse-event information was provided.

Javadipour et al. did not report attendance, adherence, exercise fidelity, withdrawals, or adverse events [22]. Therefore, the absence of reported adverse events in this study should not be interpreted as evidence that no adverse events occurred.

### 3.6. Overall Synthesis of Evidence

Formal assessment of reporting bias or small-study effects was not performed because only three studies were included and no meta-analysis was undertaken. Searches of trial registries and grey-literature sources did not identify additional eligible unpublished study reports. Nevertheless, selective reporting could not be ruled out for Jones et al. and Javadipour et al. because prospective protocols or registrations were not identified and some outcome information was incomplete or internally inconsistent. Publication bias therefore remains possible and could not be formally excluded.

## 4. Discussion

### 4.1. Principal Findings

This systematic review identified two randomized controlled trials and one controlled quasi-experimental study evaluating school-based exercise interventions for adolescents with NSLBP [20,21,22]. Across the three studies, 194 participants were allocated to intervention or control conditions, and 184 contributed to the final analyses available. All interventions lasted eight weeks and incorporated multiple exercise components, but their frequency, content, progression, supervision, and additional home components differed substantially.

The findings suggest that school-based exercise may improve some pain-related and physical outcomes, but the pattern of effects was neither uniform nor sufficient to support broad conclusions regarding intervention effectiveness. The most favorable comparative findings were reported for pain during the preceding month and three-month LBP prevalence in Fanucchi et al. [21]. Jones et al. reported a large effect size for pain intensity and reduced interference with physical activity, although a direct between-group effect estimate with a confidence interval was not provided [20]. In contrast, Fanucchi et al. found no clear between-group differences in current pain, Jones et al. found no effect on school absenteeism, and the reduction in days with LBP did not meet that study’s prespecified statistical threshold [20,21]. None of the studies directly evaluated LBP recurrence using a predefined recurrence definition.

The evidence regarding LBP-related disability was particularly limited. Jones et al. assessed interference with physical activity and school absenteeism, but these outcomes do not constitute a validated disability-specific measure [20]. Javadipour et al. used a 25-item Quebec measure that appears to represent pain-related disability, although the authors described it as pain intensity [22]. This inconsistency, together with discrepancies in the participant and outcome data, prevented confident interpretation of the construct. Consequently, the available studies do not establish whether school-based exercise improves LBP-related disability in adolescents.

### 4.2. Interpretation of Pain and Physical Outcomes

Fanucchi et al. provided the most informative comparative estimates for pain intensity [21]. Between-group differences favored exercise by 2.2 cm at three months and 2.0 cm at six months for pain experienced during the preceding month. These estimates exceeded the pediatric contextual benchmark of approximately 10 mm on a 100-mm VAS [18]. However, this threshold was derived from within-person changes in broader pediatric pain populations and was not developed to determine between-group superiority in adolescents with NSLBP. The findings should therefore be interpreted as potentially clinically relevant rather than as definitive evidence that a validated minimum important difference was achieved.

Importantly, the effect depended on the pain construct assessed. Fanucchi et al. found no clear between-group difference in current pain at either three or six months [21]. Current pain and pain recalled over the preceding month capture different aspects of the pain experience, and the discrepancy between these outcomes illustrates how recall period and outcome definition can influence the apparent intervention effect. Future studies should prespecify the pain construct and recall period and use consistent, age-appropriate measures.

Jones et al. reported a large effect size for pain intensity following the eight-week intervention [20]. Nevertheless, the absence of a directly reported between-group mean difference and confidence interval, the lack of assessor blinding, and the exclusion of participants and their matched partners after withdrawal reduce confidence in the precision of this finding. The result supports a possible beneficial effect but does not provide a sufficiently precise estimate of its magnitude.

The reductions in three-month LBP prevalence reported by Fanucchi et al. are also noteworthy, with absolute risk reductions of 24% at three months and 40% at six months [21]. However, these findings originated from a single study and should not be interpreted as evidence that exercise prevents recurrence or the development of chronic LBP. Similarly, the number of days with pain assessed by Jones et al. represented symptom frequency during a seven-day period rather than recurrence following a symptom-free interval [14].

Several improvements were observed in flexibility, mobility, muscular endurance, and other physical or motor outcomes [20,21,22]. These changes may represent exercise-specific adaptations, but they should not be interpreted as equivalent to improvements in disability or participation. In Jones et al., changes in physical outcomes were not significantly correlated with changes in pain intensity [20]. Fanucchi et al. also found no consistent effects on lumbar stability, proprioception, rectus femoris flexibility, or psychological well-being [21]. These findings do not support a single physical mechanism through which exercise reduces pain and indicate that improvements in physical performance do not necessarily explain improvements in pain-related outcomes.

Published pediatric pain studies have proposed contextual thresholds of approximately 1 point on a 0–10 numerical rating scale in adolescents with chronic pain and 10 mm on a 100-mm VAS in children with acute pain [17,18]. However, neither threshold was developed to determine between-group superiority in adolescents with NSLBP. They were therefore used only to contextualize the magnitude of the observed changes, rather than as definitive evidence that a validated minimum important difference was achieved.

### 4.3. Relationship to Previous Evidence

The present findings are broadly consistent with previous reviews suggesting that exercise may have beneficial effects for children and adolescents with LBP, while also confirming the scarcity and heterogeneity of the available evidence [3,4,6,8]. The narrower focus of the present review—exercise delivered within school settings to adolescents with existing NSLBP—resulted in a substantially smaller evidence base than reviews that included clinical, community, or home-based interventions and broader pediatric populations.

A recent meta-analysis that pooled pain-intensity data from Jones et al. and Fanucchi et al. found an uncertain overall effect of exercise compared with no intervention or usual care, accompanied by substantial heterogeneity and an extremely wide confidence interval [23]. This finding supports the decision in the present review to interpret the studies separately. Standardization through a common effect metric would not resolve the clinically relevant differences in pain definitions, recall periods, follow-up times, and intervention delivery.

The interventions included in the present review were predominantly directed toward strength, flexibility, stabilization, mobility, and posture. Contemporary recommendations for adolescents with persistent nonspecific back pain emphasize a broader biopsychosocial perspective, particularly when pain is chronic or substantially affects participation [24]. A scoping review of randomized trials similarly found that adolescent back-pain interventions have predominantly targeted biomechanical factors, with limited attention to pain beliefs, psychological factors, social context, or family involvement [25]. The interventions identified in the present review reflect this same pattern. Fanucchi et al. incorporated education and assessed psychological well-being, but none of the included programs explicitly evaluated a multidisciplinary intervention addressing physical, psychological, and social domains [21].

### 4.4. Implications for Practice and School-Based Delivery

All three interventions shared some descriptive characteristics: an eight-week duration, progressive exercise, a structured school-based component, and the inclusion of strengthening, stabilization, flexibility, mobility, or postural exercises [20,21,22]. However, these shared characteristics should not be interpreted as the active components responsible for the observed effects. No study directly compared different exercise modalities, frequencies, intensities, progression strategies, or intervention durations. The evidence therefore does not permit a recommendation regarding an optimal exercise type, frequency, intensity, session duration, or total dose.

Attendance at supervised sessions was relatively high when reported. Jones et al. reported 88% compliance, and most participants in Fanucchi et al. attended all eight supervised classes [20,21]. Adherence to the home component in Fanucchi et al. was less consistent, suggesting that supervised school delivery and unsupervised home exercise may produce different levels of engagement [21]. Nevertheless, attendance data alone do not establish feasibility. Resources, staffing requirements, scheduling, intervention fidelity, acceptability, cost, and implementation barriers were not formally evaluated.

Safety also remains uncertain. Jones et al. did not report that pain prevented participants from completing exercise sessions, and Fanucchi et al. reported one serious back injury that occurred outside the study intervention [20,21]. Javadipour et al. did not report adverse events [22]. Because adverse-event monitoring was incomplete, the absence of reported intervention-related events should not be interpreted as evidence that the programs were definitively safe.

Schools may provide a potentially valuable setting for reaching adolescents and integrating supervised exercise into existing routines. However, the available evidence supports school-based exercise only as a potentially useful management option requiring further evaluation, rather than as an established or universally feasible protocol. Exercise prescription should remain individualized [26,27] and should not replace appropriate clinical assessment, particularly when symptoms are persistent, substantially disabling, or accompanied by features suggesting a specific spinal pathology.

### 4.5. Strengths and Limitations

The strengths of this review include a predefined and registered review question, searches of multiple electronic databases and supplementary sources, independent study selection and data extraction, explicit assessment of risk of bias, and a structured narrative synthesis guided by SWiM [16]. The synthesis prioritized comparative between-group estimates and confidence intervals when available, distinguished statistical significance from clinical relevance, and considered different physical outcomes as separate constructs rather than combining them into a generic measure of function.

Several limitations should also be acknowledged. First, only three studies were included, comprising a small total sample and providing limited evidence for most outcome domains. Two studies were randomized trials, whereas Javadipour et al. used a quasi-experimental design with incompletely described allocation procedures [22]. No study was judged to be at low risk of bias across all RoB 1 domains.

Second, the participants were aged 12–15 years, and Javadipour et al. included only girls [16]. The findings therefore cannot be generalized confidently to the full 10–19-year eligibility range, to all sexes, or to adolescents in different educational and sociocultural settings. Definitions of NSLBP also differed across the studies and included recent, recurrent, and chronic symptoms.

Third, the interventions and outcomes were clinically heterogeneous. Pain constructs, recall periods, assessment times, and statistical reporting differed, while physical outcomes represented distinct constructs measured using different procedures. These differences precluded a statistically meaningful meta-analysis and prevented estimation of a pooled intervention effect [28,29].

Fourth, no study provided an unambiguous assessment of LBP-related disability using an appropriately identified and interpreted adolescent-specific instrument. Evidence was also insufficient for recurrence, quality of life, long-term participation, safety, and implementation outcomes. Follow-up beyond the immediate post-intervention period was available only in Fanucchi et al. [21].

Fifth, adherence, intervention fidelity, and adverse events were incompletely reported, particularly in Javadipour et al. [22]. Internal inconsistencies in that study’s participant description and outcome tables further limited interpretation. Selective reporting and publication bias also remain possible and could not be formally excluded.

Finally, a formal certainty-of-evidence assessment using GRADE was not undertaken, although this framework is currently considered the international standard for assessing the certainty of evidence in systematic reviews [30]. Consequently, formal certainty categories cannot be assigned to the findings, and the conclusions are not accompanied by an outcome-level rating of confidence. The small evidence base, risk-of-bias concerns, heterogeneous outcome definitions, imprecision, and incomplete reporting should therefore be considered when interpreting the conclusions.

### 4.6. Future Research

Future research should prioritize adequately powered [31], prospectively registered, multicenter randomized controlled trials with concealed allocation, blinded outcome assessment where possible, intention-to-treat analyses, and appropriately adjusted analyses for cluster-randomized designs. Studies should use clearly defined diagnostic criteria and distinguish recent, recurrent, and chronic NSLBP.

Outcome measurement should include standardized pain definitions and recall periods, validated age-appropriate disability instruments, participation, school attendance, quality of life, psychological factors, and clearly defined recurrence. Longer follow-up is required to determine whether observed improvements persist and whether school-based exercise influences subsequent episodes of LBP.

Exercise protocols should be reported using frameworks such as CERT or TIDieR, including frequency, intensity, session duration, exercise type, progression, supervision, provider qualifications, adherence, fidelity, and co-interventions. Direct comparisons or factorial designs are needed to determine whether particular exercise components or dosages provide additional benefit. Future interventions should also examine whether incorporating pain education, self-management, psychological or behavioral components, and family involvement improves patient-important outcomes.

Finally, studies conducted in schools should formally evaluate implementation outcomes, including acceptability to students and staff, accessibility, resource requirements, costs, fidelity, adverse events, and equity. These data are necessary before school-based exercise programs can be recommended as feasible and scalable strategies for adolescents with NSLBP.

## 5. Conclusions

Limited evidence from two randomized controlled trials and one controlled quasi-experimental study suggests that school-based exercise may reduce some pain-related outcomes and improve selected physical and motor outcomes in adolescents aged 12–15 years with NSLBP. The most informative comparative findings concerned pain experienced during the preceding month and three-month LBP prevalence; however, effects were not consistent across pain constructs or participation outcomes. Evidence remains insufficient regarding LBP-related disability, recurrence, long-term participation, safety, and implementation feasibility. Given the small samples, clinical and methodological heterogeneity, risk-of-bias concerns, and incomplete reporting, no specific exercise modality, frequency, intensity, duration, or dosage can currently be recommended. School-based exercise should therefore be regarded as a potentially useful management option requiring further evaluation rather than as an established standard of care. Adequately powered and prospectively registered randomized trials using standardized, age-appropriate, patient-important outcomes, longer follow-up periods, and systematic assessment of adherence, fidelity, adverse events, and implementation outcomes are required.

## Figures and Tables

**Table 1:** Characteristics and participant flow of the included studies.

Study	Country and Design	Participants and NSLBP Identification	Participant Flow	Intervention, Comparator, and Follow-Up	Outcomes
Jones et al., 2007 [20]	United Kingdom; matched-pair randomized controlled trial; two secondary schools	Adolescents in Grades 9–10 with recurrent NSLBP identified using a standardized questionnaire and confirmed by interview; analyzed sample age: 14.6 ± 0.6 years in the intervention group and 14.6 ± 0.5 years in the control group	405 screened; 88 recurrent NSLBP cases; 62 allocated (31 intervention, 31 control); four participants withdrew and four matched partners were excluded; 54 analyzed (27 per group)	Eight-week school-based exercise rehabilitation program versus normal daily activities/wait-list control; assessment immediately after eight weeks	Pain days and intensity; interference with physical activity; school absenteeism; flexibility; hip range of motion; spinal mobility; abdominal-muscle endurance
Fanucchi et al., 2009 [21]	South Africa; randomized controlled trial; two government primary schools	Students aged 12–13 years in Grades 6–7 reporting LBP during the previous three months; specific spinal, neurological, and systemic conditions excluded	99 screened; 72 randomized (39 intervention, 33 control); Month 3: 39 and 32 assessed; Month 6: 38 and 32 assessed; two losses overall; 70 assessed at final follow-up	Eight-week school-based exercise program plus home exercises versus no intervention; assessments at baseline, Month 3, and Month 6	Current and past-month pain intensity; three-month LBP prevalence; muscle flexibility; neural mobility; lumbar stability; proprioception; psychological well-being
Javadipour et al., 2021 [22]	Iran; controlled quasi-experimental pre-test–post-test study with reported random group division; schools in Lahijan	Girls aged 12–15 years with specialist-confirmed chronic NSLBP lasting more than three months; Quebec measure score >50; participants with and without hyperlordosis	60 selected and divided into four groups of 15; tables indicate 60 analyzed; attrition not reported; reported lordosis strata account for only 45 participants	Eight-week stretching and core-stability program versus no exercise program; immediate post-intervention assessment	Quebec Back Pain measure, described by the authors as pain intensity but corresponding to a disability-related construct; lumbar lordosis angle; trunk flexor, extensor, and lateral-flexor endurance; static and dynamic balance.

**Table 2:** Exercise interventions summarized according to the FITT principles.

Study	Frequency	Intensity and Progression	Time	Type	Delivery, Additional Components, and Reported Adherence
Jones et al., 2007 [20]	Two supervised group sessions/week; 16 planned sessions	Time-contingent and progressive program; standardized exercises and repetitions; all participants progressed at the same rate; specific loads and physiological intensity targets not reported	Eight weeks; approximately 30 min/session	Strength, flexibility, and aerobic exercises; pain-relieving, reconditioning, and progressive exercises	School-based; supervising professional not specified; home pain-relieving exercises encouraged; all intervention participants attended at least 12 of 16 sessions; overall compliance was 88%
Fanucchi et al., 2009 [21]	One supervised class/week; eight classes; weekly home-exercise program	Exercises progressed steadily over eight weeks; specific loading parameters not reported	Eight weeks; 40–45 min/class; initial 10–15-min educational session	Spinal and pelvic stabilization, core-muscle control, flexibility exercises, posture, and spinal-alignment education	Conducted during school hours by a registered physiotherapist; most participants attended all eight classes; five participants (13%) missed more than one class; approximately one-third performed home exercises ≥3 times/week, 20% performed them <2 times/week, and one participant performed none
Javadipour et al., 2021 [22]	Three supervised sessions/week; 24 planned sessions	Progression through reduced rest intervals and increased sets, repetitions, and contraction duration; strengthening holds progressed from 10 to 35 s; stretching repetitions progressed from 10 to 30	Eight weeks; 45 min/session	Warm-up; core-stability and lumbar-pelvic stretching exercises; abdominal, lumbar, flexor, extensor, lateral-trunk, and balance-related exercises; cool-down	Conducted in the school hall under researcher supervision; attendance, adherence, and intervention fidelity not reported

**Table 3:** Reported effects of school-based exercise interventions.

Study and Outcome	Assessment	Reported Result	Statistical Information
Jones et al. [20]: pain intensity	Post-intervention, eight weeks	Exercise change: −2.73 points; control change: 0.70 points	Exercise 95% CI −3.16 to −2.29; control 95% CI 0.04 to 1.36; interaction *p* = 0.001; ES = 1.47
Jones et al. [20]: days with LBP	Post-intervention, eight weeks	Exercise change: −0.56 days; control change: 0.22 days	Exercise 95% CI −1.06 to −0.05; control 95% CI −0.27 to 0.72; interaction *p* = 0.036; ES = 0.39
Jones et al. [20]: prevented physical activity	Post-intervention, eight weeks	Exercise change: −0.96 occasions; control change: 0.07 occasions	Exercise 95% CI −1.35 to −0.58; control 95% CI −0.24 to 0.39; interaction *p* = 0.001; ES = 0.99
Jones et al. [20]: school absenteeism	Post-intervention, eight weeks	Exercise change: −0.04 absences; control change: 0.00 absences	Exercise 95% CI −0.11 to 0.04; control 95% CI −0.15 to 0.15; interaction *p* = 0.661; ES = 0.27
Jones et al. [20]: physical outcomes	Post-intervention, eight weeks	Improvements in flexibility, hip ROM, trunk endurance, lateral flexion, and lumbar sagittal mobility	Interaction p = 0.001 for each outcome; ES range = 0.52–0.91
Fanucchi et al. [21]: pain during preceding month	Month 3	Between-group difference in change: −2.2 cm	95% CI −3.5 to −1.0
Fanucchi et al. [21]: pain during preceding month	Month 6	Between-group difference in change: −2.0 cm	95% CI −3.5 to −0.5
Fanucchi et al. [21]: current pain	Months 3 and 6	Between-group differences: −1.2 cm and 0.3 cm	Month 3: 95% CI −2.6 to 0.2; Month 6: 95% CI −1.3 to 1.9
Fanucchi et al. [21]: three-month LBP prevalence	Months 3 and 6	Absolute risk reductions: 24% and 40%	Month 3: 95% CI 4% to 41%; Month 6: 95% CI 18% to 57%
Fanucchi et al. [21]: flexibility and neural mobility	Month 3	Between-group differences favored exercise for hamstrings, iliopsoas, and passive straight-leg raise	Differences ranged from 6° to 16°; reported 95% CIs excluded the null
Fanucchi et al. [21]: lumbar stability, rectus femoris flexibility, proprioception, and well-being	Months 3 and 6	No clear between-group differences	Reported 95% CIs included the null or between-group analyses were not statistically significant
Javadipour et al. [16]: Quebec measure	Post-intervention, eight weeks	Reductions reported in exercise groups with and without hyperlordosis	Between-group *p* = 0.001 for both strata; effect estimates and 95% CIs not reported
Javadipour et al. [22]: lordosis and trunk endurance	Post-intervention, eight weeks	Between-group differences reported for lordosis angle and flexor, extensor, and lateral-flexor endurance	Reported *p* ≤ 0.016; effect estimates and 95% CIs not reported
Javadipour et al. [22]: static and dynamic balance	Post-intervention, eight weeks	Differences reported only in participants without hyperlordosis	Without hyperlordosis: *p* = 0.001; with hyperlordosis: *p* = 0.477 and *p* = 0.541

Abbreviations: CI, confidence interval; ES, effect size; LBP, low back pain; ROM, range of motion. For Fanucchi et al., between-group estimates represent the difference between changes from baseline in the exercise and control groups. Jones et al. did not report direct between-group mean differences; therefore, group-specific changes and group-by-time interaction results are presented. Javadipour et al. did not provide confidence intervals, and additional effects were not reconstructed because of internal reporting inconsistencies.

## Data Availability

No new data were created or analyzed in this study. Data sharing is not applicable to this article.

## Supplementary Materials

The following supporting information can be downloaded at: https://www.mdpi.com/article/10.3390/medsci14040473/s1, Supplementary Table S1: Electronic databases and complete search strategies used for the systematic literature search, including search terms, Boolean operators, database-specific syntax, search dates, and the number of records retrieved from PubMed/MEDLINE, Scopus, Web of Science, Cochrane CENTRAL, PEDro, and ScienceDirect; Supplementary Table S2: Additional Sources, Grey Literature, Trial Registries, and Citation Searching; Supplementary Table S3: Reasons for exclusion of full-text studies assessed for eligibility, including the frequency of each exclusion criterion; File S1: PRISMA_2020_checklist.

## Abbreviations

The following abbreviations are used in this manuscript:

Abbreviation	Definition
CENTRAL	Cochrane Central Register of Controlled Trials
CERT	Consensus on Exercise Reporting Template
CI	confidence interval
df	degrees of freedom
ES	effect size
FITT	frequency, intensity, time, and type
GRADE	Grading of Recommendations Assessment, Development and Evaluation
ICTRP	International Clinical Trials Registry Platform
LBP	low back pain
MEDLINE	Medical Literature Analysis and Retrieval System Online
MeSH	Medical Subject Headings
NSLBP	nonspecific low back pain
PE	physical education
PEDro	Physiotherapy Evidence Database
PICOT	population, intervention, comparator, outcome, and time
PRISMA	Preferred Reporting Items for Systematic Reviews and Meta-Analyses
PRISMA-P	Preferred Reporting Items for Systematic Review and Meta-Analysis Protocols
PROSPERO	International Prospective Register of Systematic Reviews
RoB 1	Cochrane Risk of Bias tool, version 1
ROBINS-I	Risk of Bias in Non-randomized Studies of Interventions
ROM	range of motion
SWiM	Synthesis Without Meta-analysis
TIDieR	Template for Intervention Description and Replication
VAS	visual analogue scale
WHO	World Health Organization
ASLR	Active Straight Leg Raise
