# Supplementary material for: Exercise-Based Interventions for the Management of Low Back Pain in Adolescents in School Settings: A Systematic Review
Source: Med Sci (Basel). 2026 Aug 12;14(4):473. doi: 10.3390/medsci14040473 (PMC13515185; doi:10.3390/medsci14040473)
Supplement: Supplementary file 1 [file medsci-14-00473-s001.zip › medsci-4447194-supplementary.pdf]

Supplementary: Electronic Databases and Search Strategies

Supplementary Table S1. Electronic databases and complete search strategies used for the systematic literature search, including search terms, Boolean operators, database-specific syntax, search dates, and the number of records retrieved from PubMed/MEDLINE, Scopus, Web of Science, Cochrane CENTRAL, PEDro, and ScienceDirect.

The systematic search was conducted using controlled vocabulary terms (Medical Subject Headings [MeSH], when applicable) and free-text terms related to four main concepts: **low back pain**, **adolescents**, **exercise-based interventions**, and **school settings**. Boolean operators AND and OR were used to combine the search terms. The search strategies were adapted according to the syntax and indexing system of each electronic database.

The electronic databases searched were PubMed/MEDLINE, Scopus, Web of Science, Cochrane Central Register of Controlled Trials (CENTRAL), PEDro, and ScienceDirect. The final search was conducted on **21 May 2026**. No restrictions regarding language, country, or publication date were applied.

PubMed/MEDLINE

Date of last search: 21 May 2026
Filters or limits: None
Records identified: 1,425

**Concept**	**Search terms**
**Condition (Low Back Pain)**	"Low Back Pain"[Mesh] OR "low back pain" OR lumbago OR lumbalgia OR "lumbar pain" OR "back pain" OR "nonspecific low back pain" OR "non-specific low back pain" OR NSLBP
**Population (Adolescents)**	"Adolescent"[Mesh] OR "Child"[Mesh] OR adolescent* OR adolescenc* OR teenager* OR teen* OR youth OR student* OR schoolchild* OR "school-aged"
**Intervention (Exercise)**	"Exercise Therapy"[Mesh] OR "Exercise"[Mesh] OR "Motor Activity"[Mesh] OR exercis* OR "therapeutic exercise" OR "physical activity" OR strengthening OR "resistance training" OR "stabilization exercise" OR "stabilization training" OR "core training" OR Pilates OR stretching OR "flexibility exercise" OR "motor control exercise" OR "postural exercise" OR rehabilitation OR physiotherap* OR kinesiotherap* OR "training program*"
**Setting (School)**	"Schools"[Mesh] OR "School Health"[Mesh] OR school* OR "school-based" OR "school setting" OR classroom OR "physical education" OR PE

Complete search strategy:

(("Low Back Pain"[Mesh] OR "low back pain" OR lumbago OR lumbalgia OR "lumbar pain" OR "back pain" OR "nonspecific low back pain" OR "non-specific low back pain" OR NSLBP) AND ("Adolescent"[Mesh] OR "Child"[Mesh] OR adolescent* OR adolescenc* OR teenager* OR teen* OR youth OR student* OR schoolchild* OR "school-aged") AND ("Exercise Therapy"[Mesh] OR "Exercise"[Mesh] OR "Motor Activity"[Mesh] OR exercis* OR "therapeutic exercise" OR "physical activity" OR strengthening OR "resistance training" OR "stabilization exercise" OR "stabilization training" OR "core training" OR Pilates OR stretching OR "flexibility exercise" OR "motor control

exercise" OR "postural exercise" OR rehabilitation OR physiotherap* OR kinesiotherap* OR "training program*") AND ("Schools"[Mesh] OR "School Health"[Mesh] OR school* OR "school-based" OR "school setting" OR classroom OR "physical education" OR PE))

Total: 1,425 records

Scopus

Date of last search: 21 May 2026
Search field: Title, Abstract, and Keywords (TITLE-ABS-KEY)
Filters or limits: None
Records identified: 128

Concept	Search terms
**Condition**	"low back pain" OR lumbago OR lumbalgia OR "lumbar pain" OR "back pain" OR "nonspecific low back pain" OR "non-specific low back pain" OR NSLBP
**Population**	adolescent* OR adolescenc* OR teenager* OR teen* OR youth OR student* OR schoolchild* OR "school-aged"
**Intervention**	exercis* OR "therapeutic exercise" OR "exercise therapy" OR "physical activity" OR strengthening OR "resistance training" OR "stabilization exercise" OR "stabilization training" OR "core training" OR Pilates OR stretching OR "flexibility exercise" OR "motor control exercise" OR "postural exercise" OR rehabilitation OR physiotherap* OR kinesiotherap* OR "training program*"
**Setting**	school* OR "school-based" OR "school setting" OR classroom OR "physical education"

Complete search strategy:

TITLE-ABS-KEY ("low back pain" OR lumbago OR lumbalgia OR "lumbar pain" OR "back pain" OR "nonspecific low back pain" OR "non-specific low back pain" OR NSLBP)
AND TITLE-ABS-KEY (adolescent* OR adolescenc* OR teenager* OR teen* OR youth OR student* OR schoolchild* OR "school-aged")
AND TITLE-ABS-KEY (exercis* OR "therapeutic exercise" OR "exercise therapy" OR "physical activity" OR strengthening OR "resistance training" OR "stabilization exercise" OR "stabilization training" OR "core training" OR Pilates OR stretching OR "flexibility exercise" OR "motor control exercise" OR "postural exercise" OR rehabilitation OR physiotherap* OR kinesiotherap* OR "training program*")
AND TITLE-ABS-KEY (school* OR "school-based" OR "school setting" OR classroom OR "physical education")

Total: 128 records

Web of Science

Date of last search: 21 May 2026
Search field: Topic (TS)
Timespan: All years

Filters or limits: None
Records identified: 79

Search set	Strategy
**#1 – Condition**	TS=("low back pain" OR lumbago OR lumbalgia OR "lumbar pain" OR "back pain" OR "nonspecific low back pain" OR "non-specific low back pain" OR NSLBP)
**#2 – Population**	TS=(adolescent* OR adolescenc* OR teenager* OR teen* OR youth OR student* OR schoolchild* OR "school-aged")
**#3 – Intervention**	TS=(exercis* OR "therapeutic exercise" OR "exercise therapy" OR "physical activity" OR strengthening OR "resistance training" OR "stabilization exercise" OR "stabilization training" OR "core training" OR Pilates OR stretching OR "flexibility exercise" OR "motor control exercise" OR "postural exercise" OR rehabilitation OR physiotherap* OR kinesiotherap* OR "training program*")
**#4 – Setting**	TS=(school* OR "school-based" OR "school setting" OR classroom OR "physical education")
**#5 – Final search**	#1 AND #2 AND #3 AND #4

Complete search strategy:

TS=("low back pain" OR lumbago OR lumbalgia OR "lumbar pain" OR "back pain" OR "nonspecific low back pain" OR "non-specific low back pain" OR NSLBP) AND TS=(adolescent* OR adolescenc* OR teenager* OR teen* OR youth OR student* OR schoolchild* OR "school-aged") AND TS=(exercis* OR "therapeutic exercise" OR "exercise therapy" OR "physical activity" OR strengthening OR "resistance training" OR "stabilization exercise" OR "stabilization training" OR "core training" OR Pilates OR stretching OR "flexibility exercise" OR "motor control exercise" OR "postural exercise" OR rehabilitation OR physiotherap* OR kinesiotherap* OR "training program*") AND TS=(school* OR "school-based" OR "school setting" OR classroom OR "physical education")

Total: 79 records

Cochrane Central Register of Controlled Trials (CENTRAL)

Date of last search: 21 May 2026
Filters or limits: None
Records identified: 80

Condition

#1 MeSH descriptor: [Low Back Pain] explode all trees
#2 ("low back pain"):ti,ab,kw
#3 (lumbago):ti,ab,kw
#4 (lumbalgia):ti,ab,kw
#5 ("lumbar pain"):ti,ab,kw
#6 ("back pain"):ti,ab,kw
#7 ("nonspecific low back pain"):ti,ab,kw

#8 ("non-specific low back pain"):ti,ab,kw
#9 (NSLBP):ti,ab,kw

Population

#10 MeSH descriptor: [Adolescent] explode all trees
#11 MeSH descriptor: [Child] explode all trees
#12 (adolescent*):ti,ab,kw
#13 (adolescenc*):ti,ab,kw
#14 (teenager*):ti,ab,kw
#15 (teen*):ti,ab,kw
#16 (youth):ti,ab,kw
#17 (student*):ti,ab,kw
#18 (schoolchild*):ti,ab,kw
#19 ("school-aged"):ti,ab,kw

Intervention

#20 MeSH descriptor: [Exercise Therapy] explode all trees
#21 MeSH descriptor: [Exercise] explode all trees
#22 MeSH descriptor: [Motor Activity] explode all trees
#23 (exercis*):ti,ab,kw
#24 ("therapeutic exercise"):ti,ab,kw
#25 ("physical activity"):ti,ab,kw
#26 (strengthening):ti,ab,kw
#27 ("resistance training"):ti,ab,kw
#28 ("stabilization exercise"):ti,ab,kw
#29 ("stabilization training"):ti,ab,kw
#30 ("core training"):ti,ab,kw
#31 (Pilates):ti,ab,kw
#32 (stretching):ti,ab,kw
#33 ("flexibility exercise"):ti,ab,kw
#34 ("motor control exercise"):ti,ab,kw
#35 ("postural exercise"):ti,ab,kw
#36 (rehabilitation):ti,ab,kw
#37 (physiotherap*):ti,ab,kw
#38 (kinesiotherap*):ti,ab,kw
#39 ("training program*"):ti,ab,kw

Setting

#40 MeSH descriptor: [Schools] explode all trees
#41 MeSH descriptor: [School Health] explode all trees
#42 (school*):ti,ab,kw
#43 ("school-based"):ti,ab,kw
#44 ("school setting"):ti,ab,kw
#45 (classroom):ti,ab,kw
#46 ("physical education"):ti,ab,kw

Final search:

(#1 OR #2 OR #3 OR #4 OR #5 OR #6 OR #7 OR #8 OR #9)
AND (#10 OR #11 OR #12 OR #13 OR #14 OR #15 OR #16 OR #17 OR #18 OR #19)
AND (#20 OR #21 OR #22 OR #23 OR #24 OR #25 OR #26 OR #27 OR #28 OR #29 OR #30 OR #31 OR #32 OR #33 OR #34 OR #35 OR #36 OR #37 OR #38 OR #39)
AND (#40 OR #41 OR #42 OR #43 OR #44 OR #45 OR #46)

Total: 80 records

PEDro (Physiotherapy Evidence Database)

Date of last search: 21 May 2026
Filters or limits: No date restriction
Records identified: 28

Because PEDro provides a more restricted search interface than bibliographic databases, combinations of simplified searches were used based on the main concepts of the review.

Search terms/combinations:

"low back pain" adolescent exercise

"low back pain" school exercise

"low back pain" adolescent rehabilitation

"low back pain" student exercise

"back pain" adolescent school

"lumbar pain" adolescent exercise

Total records identified after combining the searches and removing duplicates within the database: 28

ScienceDirect

Date of last search: 21 May 2026
Filters or limits: None
Records identified: 201

Search concepts:

**Concept**	**Search terms**
**Condition**	"low back pain" OR lumbago OR lumbalgia OR "lumbar pain" OR "back pain" OR "nonspecific low back pain" OR "non-specific low back pain"
**Population**	adolescent OR adolescents OR teenager OR teenagers OR youth OR student OR students OR schoolchild
**Intervention**	exercise OR "therapeutic exercise" OR "exercise therapy" OR "physical activity" OR strengthening OR "resistance training" OR stabilization OR "core training" OR Pilates

	OR stretching OR "motor control exercise" OR rehabilitation OR physiotherapy
**Setting**	school OR schools OR "school-based" OR "school setting" OR classroom OR "physical education"

Reconstructed complete search strategy:

("low back pain" OR lumbago OR lumbalgia OR "lumbar pain" OR "back pain" OR "nonspecific low back pain" OR "non-specific low back pain") AND (adolescent OR adolescents OR teenager OR teenagers OR youth OR student OR students OR schoolchild) AND (exercise OR "therapeutic exercise" OR "exercise therapy" OR "physical activity" OR strengthening OR "resistance training" OR stabilization OR "core training" OR Pilates OR stretching OR "motor control exercise" OR rehabilitation OR physiotherapy) AND (school OR schools OR "school-based" OR "school setting" OR classroom OR "physical education")

Total: 201 records

Supplementary Table S2. Additional Sources, Grey Literature, Trial Registries, and Citation Searching.

To maximize search sensitivity and reduce the potential impact of publication bias, complementary searches were conducted in grey literature sources, clinical trial registries, and other sources. The final complementary searches were conducted on **21 May 2026**.

Source	Search approach	Date of last search
**Google Scholar**	Combinations of the terms "low back pain", adolescent*, exercise, "exercise therapy", school*, and "school-based"	21 May 2026
**OpenGrey**	Combinations of "low back pain" AND adolescent* AND exercise	21 May 2026
**ProQuest Dissertations & Theses**	Combinations of "low back pain" AND (adolescent* OR teenager* OR youth) AND (exercise OR "exercise therapy") AND (school* OR "school-based")	21 May 2026
**ClinicalTrials.gov**	Condition/disease: Low Back Pain; Other terms: adolescent, exercise, school	21 May 2026
**WHO ICTRP**	"low back pain" AND adolescent* AND exercise	21 May 2026
**Reference lists of included studies**	Reference lists of all studies meeting the eligibility criteria were manually screened for potentially eligible studies	21 May 2026
**Relevant systematic reviews**	Reference lists of relevant systematic reviews on low back pain and conservative or exercise-based interventions in children and adolescents were manually screened	21 May 2026
**Citation tracking**	Forward and backward citation searching was conducted for eligible studies to identify additional potentially relevant	21 May

	reports	2026

Supplementary Table S3. Reasons for exclusion of full-text studies assessed for eligibility, including the frequency of each exclusion criterion.

**Motivo da Exclusão**	**Frequência (n)**
Population not eligible	9
No low back pain diagnosis or outcome	7
Intervention not exercise-based	5
Not conducted in school setting	9
Wrong study design	5
Prevention studies only	5
No appropriate comparator	3
**Total**	**43**
